# The influence of media narratives on microplastics risk perception

**DOI:** 10.7717/peerj.16338

**Published:** 2023-11-02

**Authors:** Valeria Pop, Alexandru Ozunu, Dacinia Crina Petrescu, Adrian-Daniel Stan, Ruxandra Malina Petrescu-Mag

**Affiliations:** 1 Research Institute for Sustainability and Disaster Management Based on High-Performance Computing, Faculty of Environmental Science and Engineering, Babes-Bolyai University, Cluj-Napoca, Romania; 2 Disaster Management Training and Education Centre for Africa (DiMTEC), University of the Free State, Bloemfontein, South Africa; 3 Department of Hospitality Services, Faculty of Business, Babes-Bolyai University, Cluj-Napoca, Cluj, Romania; 4 Department of Economy and Rural Development, Faculty of Gembloux Agro-Bio Tech, University of Liège, Gembloux, Belgium; 5 Department of International Studies and Contemporary History, Faculty of History and Philosophy, Babes-Bolyai University, Cluj-Napoca, Cluj, Romania; 6 Doctoral School “International Relations and Security Studies”, Babes-Bolyai University, Cluj-Napoca, Romania

**Keywords:** Awareness, Microplastics, Health, Environment, Social media

## Abstract

**Background:**

Media are the interface between scientists and citizens, communicating and interpreting the risk message and powerfully influencing individual awareness, public debate, and, hence, people’s behavior. Pollution by microplastics (MPs), a threat to public health and terrestrial and marine ecosystems, has received research, media, and public interest. However, how MPs environmental and health risks are reflected in the media and assessed in the scientific literature does not find consensus over time. To date, few studies have examined social aspects around MPs, such as, for example, factors that influence awareness and perception of the risk of MPs. In this context, the objective of this study is twofold. First, we determined if media narratives influenced Romanians’ awareness of MPs, and second, we investigated if media narratives influenced Romanians’ perceptions of MPs health and environmental risk.

**Method:**

An online survey was conducted among 417 Romanian respondents. The questionnaire had 21 questions. The questions were related to the awareness of MPs, the perceived health risk of MPs, the perceived environmental risk of MPs, the intensity of exposure to media narratives about the MPs impact on health and the environment, and the demographics. Binary logistic regression was run to identify what media narratives influenced MPs awareness and risk perception. In recent times, mass media has shaped perceptions of health and environmental risks, driven by events like COVID-19 and global climate change. Our study relies on media narratives as its foundation.

**Results:**

Binary logistic regression showed that the awareness of MPs is influenced by the media narrative “Microplastics in the sea threaten fish stocks” (*p* = 0.001). When the frequency of exposure to this media narrative increases, the probability of reporting awareness of MPs increases. Likewise, an increase in age represents a higher probability of reporting awareness of MPs. The perceived health risk of MPs, with the highest weighting, was related to the dependent variable “Leakage of harmful chemicals from MPs affects the soil” (*p* = 0.014).

**Conclusions:**

Media narratives about plastic and MPs pollution have increased over time, influencing the perception of this risk. The study argues the need for accurate and balanced media reporting on MPs to prevent the spread of misinformation and ensure that people clearly understand MPs risks. Furthermore, a closer examination of people’s perceptions supports the design of appropriate interventions to reduce plastic consumption, thereby decreasing the risks of MPs pollution with benefits for human health and the environment.

## Introduction

The influence of the media on environmental and health risk perceptions has received increasing attention in recent years due to events that have already become part of our daily lives, such as climate change or COVID-19 (Stoddart et al., 2023). If we look at Boykoff & Boykoff’s (2007) paper, mainstream media communicated one-way messages through newspapers and television, which were, 15 years ago, one of the most powerful means of shaping and disseminating information. Today, to stay informed, we focus on social networks that provide extensive online interpersonal interactions (Liu et al., 2021; Li & Zheng, 2022). Therefore, due to the effects of social media use, mass media becomes a “social amplifier station” for the better or worse events we face every day (Oh, Lee & Han, 2021), and even a “validator” of science, disseminating the information taken from different scientific sources, to the receiving public (Gamson, 1999). Additionally, recent technological advancements, coupled with the influence of social networks, have transformed mass media into a platform for magnifying social influence.

An overstated criticism of journalists is that they are “too unquestioning” (Kitzinger, 1999) about scientific findings. Scientific discoveries and reports require translation into terms accessible to the general public, a role that the mass media must play (Weingart, Engels & Pansegrau, 2000). Additionally, scientists and the audience frequently criticized the self-inflating media coverage (Vasterman, 2005), the so-called media hype. This “media hype” is described as the first impulse that leads more to the dramatization of the news in the first place and, in the second place, to a change in the public’s perception of the type of risk presented. Research indicates that the media primarily focuses on the environmental damage of specific events and dangers and, ultimately, on opportunities, benefits, and positive outcomes (Welzenbach-Vogel et al., 2022). There is a relationship between dramatization and the predilection for novelty, on which the mass media is based and which, over time, influences people’s behaviors and attitudes, whether related to the environment in general, human health, or any other type of news that may represent a risk (Wilkins & Patterson, 1987). In addition to communicating the risk message, the media interprets it (Snyder & Rouse, 1995; Niu et al., 2022), becoming the interface between scientists and citizens, influencing individual awareness and public debate (Petrescu-Mag et al., 2022). Consequently, the media are a relevant determinant of public perceptions of risk.

### Background

An example of highly mediatized environmental and health issues is microplastics (MPs). MPs (plastic particles smaller than 5 mm) pollute the soil, air, and water due to inadequate production, consumption, and waste management (Rist et al., 2018). The source of MPs origin is an important indicator in classifying MPs particles. These can come from the fragmentation of large plastics (secondary MPs) and (primary MPs) intentionally manufactured to be put into cosmetics, paints, fertilizers, detergents, and some cleaning products. Biodegradation, thermal degradation, photodegradation, physical damage, thermo-oxidative degradation, and hydrolysis are some of the mechanisms that degrade plastic polymers and transform them into MPs (Wu et al., 2019). The shape is another indicator used to classify MPs. MPs have a wide range of shapes (fragment, paint, pellet, sphere, fiber, film, line, bead, flake, sheet, granule, *etc*., Koelmans et al., 2019; Campanale et al., 2020; Zhang et al., 2020).

According to the United Nations Environment Program (United Nations Environment Program (UNEP), 2014), one of the ten emerging environmental and human health problems facing humanity today is MPs particles. The occurrence and accumulation, but not least, the persistence of MPs in terrestrial and marine ecosystems, have become a major global concern. This problem of plastics has been recognized by the United Nations (UN) through the Sustainable Development Goals (SDGs), more precisely by Goal 14-Conservation and sustainable use of oceans, seas, and marine resources for sustainable development (Walker, 2021).

In the 1970s, the first documented report of MPs in marine ecosystems was published by Carpenter & Smith (1972). According to Naik et al. (2019), it is estimated that between 15 and 51 trillion particles of MP/NPs (93 to 236 thousand metric tons) have been found in the oceans. Car tires and brake systems are responsible for between 5% and 10% of plastic pollution in the world’s oceans. PM 2.5 in the air has a percentage between 3% and 7% (Evangeliou et al., 2020). Hale et al. (2020) specifies that each person in the United States generates 4.7 kg MPs of tire wear per year, equating to 1.8 million metric tons/per year. Due to their high availability in aquatic and terrestrial environments, MPs enter the food chain, being observed in honey, beer, salt, sugar, tea bags, milk, salmon, seaweed, shrimp, *etc*. (Santillo, Miller & Johnston, 2017; Cox et al., 2019; Markic et al., 2020; Zhang et al., 2020).

However, the demand for plastic is growing exponentially and production trends are expected to quadruple by 2050 (Suaria et al., 2016). That is why, in recent years, considerable efforts have been made to understand the route of contamination with MPs particles, and strategies are being created to remove MPs. According to Zhou et al. (2023), the main problems related to the treatment and research of MPs are, firstly, the lack of a precise definition of what constitutes MPs, which makes it challenging to perform a comparative analysis of the findings of various investigated, and secondly, the difficulty of comparing the results due to the varied methodologies used in MPs studies.

Due to the scientific evidence and the mediatization of the impact of MPs on health and the environment, there is growing concern about it. MPs research has grown exponentially in the last 5 years (Wagner & Lambert, 2018; Völker, Kramm & Wagner, 2020; Kramm et al., 2022), but the focus on environmental issues has led to an interest in research in this area since 2006, the term MPs being used for the first time in 2004 and the first research in the field dating from the early 1970s (Klingelhöfer et al., 2020).

However, how the MPs environmental and human health risks were reflected in the media and assessed in the scientific literature did not find consensus over time. Although there is much scientific evidence on the risks posed by MPs (Xiang et al., 2022; Yusuf et al., 2022), many voices still argue the contrary (Xu et al., 2022; Shi et al., 2022; Nithin et al., 2022). According to Völker, Kramm & Wagner (2020), environmental risks related to MPs are presented as hypothetical or uncertain in 67% (low-risk degree); In more than 24% of scientific studies, environmental risks are presented as stable or real (higher degree of risk). This polemic nicely commented on by Backhaus & Wagner (2020) has in one corner the null risk camp (Burton & Cervi, 2019) and in the opposite corner, the all risk camp (Rochman et al., 2015). It is from this contrast that MPs are worth our attention since it sheds light on how science frames environmental risks and how the media communicate with MPs to inform the public and, eventually, shape public opinion (Altheide, 1997; Allan, Adam & Carter, 2000; Jönsson, 2011).

Kramm et al. (2022) acknowledge a contradiction between scientific evidence, public opinion, and media coverage related to MPs. Schönbauer & Müller (2021) consider that the high degree of uncertainty of these new forms of environmental risk can explain this inconsistency of positions. This is precisely why solutions to the plastic problem should consider perceptions, attitudes, and behaviors. Previous studies show that the perception of environmental risk impacts people’s attitudes and behaviors; the higher the perception of environmental risk, the higher the response behaviors (Glaser, 2012), so there is a causal relationship between higher perception and more behavior. In this context, research should focus more on the perspectives that media, scientific knowledge, and public opinion reflect on MPs. The perception of risk related to MPs is influenced by the frequency of information published by the media and how it is presented (Wahlberg & Sjoberg, 2000; Bodemer & Gaissmaier, 2015). This risk is usually amplified by various channels, such as radio and television, but to a large extent, it is amplified by the social behavior (Renn, 2008). According to Stanton et al. (2020), media coverage has dramatized MPs, being criticized for not drawing the public’s attention to much more critical environmental issues and with a higher risk than MPs. However, the Evidence Assessment Report (SAPEA, 2019) acknowledges that despite the unknowns about MPs health and environmental impact, plastic pollution will continue to increase media attention, and the need to consider the role of social and behavioral sciences (*e.g*., on risk perception, media coverage, and narratives) when analyzing the natural sciences evidence (*e.g*., on risks, resource depletion).

### Aim and objectives of the study

To date, the factors that influence the individual perception of the risk of MPs are relatively unreported in the scientific literature (Pahl & Wyles, 2017; Yoon, Jeong & Chon, 2021; Garcia-Vazquez & Garcia-Ael, 2021; Deng et al., 2022). There are several studies on risk perception factors, such as Kramm et al. (2022), showing that individual risk perception varies according to socio-demographic factors and is determined by media reports and, not least, by environmental awareness. Renn (1991) understood risk perception as a product of social experience, which depended on people’s knowledge, self-control, and experience. Omoyajowo et al. (2021) argue that civil society actors, researchers, industry, and political decision-makers worldwide should consider the important role of the mass media in establishing the perception of the risk of MPs particles for a reduction in the use of plastic materials and not least the number of MPs emissions (Syberg et al., 2018; Mahmod et al., 2022; Janzik et al., 2023). Niu et al. (2022) highlighted that the relationship between media consumption and perceived risk varied between countries, media type, and nature of risk. Similarly, results that reported perceptions, behaviors, and attitudes toward MPs from different countries cannot be generalized to other populations. In this context, the objective of this study is twofold:

(1) First, we determined whether media narratives influenced Romanians’ awareness of MPs.

(2) Second, we investigated whether media narratives influenced Romanians’ perceptions of MPs health and environmental risk. The study fills in the gaps in social research by revealing how media narratives influence the degree of awareness of MPs, the perceived risk of these particles, and, finally, how these narratives influence the perception of their impact on the environment.

To show whether media narratives influence the perception of risk to human health and the environment and whether these narratives influence the degree of awareness of MPs particles, we developed the hypotheses described below.

### Media narratives of MPs influence the awareness of MPs

Much research has focused on the power of the media in influencing public awareness and political interventions (Cottle, 2009; Ho, Scheufele & Corley, 2013; Chapman et al., 2014; Karasneh et al., 2021). The role of media narratives and environmental awareness in predicting the risk perception of MPs was also reported in the scientific literature (Henderson & Green, 2020; Kramm et al., 2022). For example, when examining the awareness of MPs of personal care products (*e.g*., toothpaste, soap) in three groups (university students, environmental activists, and trainee beauticians) from South England, Anderson et al. (2016) found that students are less aware of MPs than environmentalists. Furthermore, a study focused on Norway showed that knowledge of the sources of MPs was low, and few respondents mentioned potential ways to solve that problem (Felipe-Rodriguez, Böhm & Doran, 2022), although Norway (which has the longest coastline in Europe) is actively involved in fighting marine litter (Lusher et al., 2021).

In addition to media narratives (reflected in TV shows, press, *etc*.), cinemas and documentaries are powerful vehicles that could contribute towards a more aware, self-reflective, and self-concerned public of MPs (Merdhi & Imanjaya, 2022). Schönbauer & Müller (2021) concluded that media narratives about MPs influenced public awareness and argued that media cycles of asserting and disputing MPs risks could undermine public confidence in scientific findings. However, as suggested by others, such as Thiele & Hudson (2021), there seems to be a personal perception bias in people with professional expertise on MPs risk, and, in this case, the influence of the media is reduced. Media narratives in the form of textual, visual, and video posts, even if sometimes they seem superficial and show an amateur level, contribute to motivating constructive behaviors and raising awareness (Hui-Jew, 2020). This type of social messaging leads to the creation of public awareness, and when used actively and repeatedly, it can direct people toward pro-environmental actions.

### Media narratives of MPs influence the perceived health risks of MPs

Plastic and MPs pollution has an upward curve and is a global concern due to the potential impact on human health. Studies (Zettler, Mincer & Amaral-Zettler, 2013; Keswani et al., 2016) show that plastics become a reservoir for pathogens and a habitat for microbial communities due to their physical properties. There is worldwide concern about human exposure, particularly to MPs. However, some scientists argued that the risks of ingestion and inhalation of MPs are relatively small, and so the health risk is Karami et al. (2018), which, of course, requires standardized analytical methods for the detection and characterization of MPs (Zhang et al., 2020).

Despite these opinions, at the United Nations Environment Assembly-5 (2022), an agreement was reached to start negotiations for a binding agreement that could represent the foundation for plastics regulation considering the life cycle (Tiller, Booth & Cowan, 2022). In November 2022, we interrogated the European Commission (on the Europe Direct network, registration number 2175406) on the EU’s directions toward combating MPs. The response emphasized, on the one hand, the need to implement economic incentives for companies to reduce MPs presence. On the other hand, the urgency to cover MPs comprehensively by the EU legislation was highlighted, even if there are several specific hard and soft law instruments with partial objectives that address the MPs in the environment (*e.g*., Marine Strategy Framework Directive, Fertilizing Products Regulation, REACH restriction proposal regarding the limitation of microparticles of synthetic polymers on the market, Plastics strategy, Waste Framework Directive, Eco-design Directive, Directive on air quality, Regulation on tire labeling). A step forward was made when the European Commission requested the European Chemicals Agency (European Chemicals Agency (ECHA), 2019) to prepare a file on the use of MPs intentionally added to professional and non-professional use products. Furthermore, the European Commission announced (as part of the European Green Economy Action Plan and the new Circular Economy Action Plan) a new initiative to tackle the unintentional release of MPs into the environment. It targets, for example, labeling, standardization, certification, and regulatory measures for accidental releases of MPs, and harmonization of the methods for measuring unintentionally released MPs, mainly from tires and textiles.

Concerns about MPs potential environmental and health effects have led to increased research and media reporting. In their study, Welzenbach-Vogel et al. (2022) indicated that journalists usually report on specific events and hazards and focus primarily on harm in terms of plastic materials. In this way, the media communicate damage first and opportunities second, thus increasing the perceived risk of MPs by individuals for their health. Catarino et al. (2021) considered that scientific communications made by the mass media should convey and contextualize scientific findings precisely to inform and not alarm the public about human health risks caused by MPs. Research has shown that communication of uncertainty does not reduce trust in the communicator (Van der Bles et al., 2022). However, it can increase the risk related to public perception of a particular topic, whether related to human health or the environment in general.

Wu et al. (2023) selected the most popular Chinese social media platform, collected 6 years of online dissemination data (2015–2020), and comprehensively analyzed public attention, attitudes, and opinions about MPs, demonstrating that more attention has been given to MPs pollution in marine environments and human health issues (*e.g*., food and drinking water). Last but not least, through the consecutive broadcasting of posts related to MPs in the previous 5 years, Chinese platforms, through this online propagation, have increased the public’s concern about the health problems of MPs.

### Media narratives of MPs influence the perceived environmental risks of MPs

Media narratives about plastic pollution have increased over time, influencing the perception of this risk. Photos and videos that illustrate marine animals suffering from plastic entanglement and ingestion helped raise our awareness. An example is a widely distributed photo of a sea turtle with a plastic straw in its nose. The case received millions of views on YouTube and was judged as a reference point that influenced the political agenda of the international community toward more synergistic cooperation to combat marine plastics pollution (Tiller, Booth & Cowan, 2022).

According to Guan et al. (2022), a short environmental video can effectively engage viewers in combating the overproduction of single-use plastic products. This support was provided by increasing risk perception, generating adverse effects, encouraging information search, and promoting pro-environmental civic actions. For example, the B.B.C. documentary series broadcast 2 years after David Attenborough’s Blue Planet II (a documentary about marine life) highlighted the amount of plastic waste in the ocean. Erik Solheim, the former head of the United Nations Environment Program, said that the documentary “helped spur a wave of action” globally (SAPEA, 2019). The “Blue Planet effect” was associated with campaigns and ads demanding regulations to reduce single-use plastics (Pahl, Wyles & Thompson, 2017; Hartley et al., 2018).

In the opposite camp, as the editor-in-chief of one of the leading environmental toxicology journals and professor at the University of Michigan, Burton & Cervi (2019) said that it was troubling that studies on MPs, which declared a severe threat to the environment, continued to be published in high-quality journals. Scientific studies are quickly picked up by the media and serve to misinform the public and decision-makers. He believed that the environmental risk of MPs (he mainly referred to microbeads) was overstated, and much more should have been done to analyze the real exposure relationships to adverse effects.

## Materials and Methods

### Ethics statement

All work reported in this article was approved by the Scientific Council of Babeș-Bolyai University of Cluj-Napoca, Romania, under the Ethics Approval no. 1958/14.02.2023.

### Data availability

The raw data from participants are downloadable as Supporting Information.

### Participants and study design

**Figure 1:**
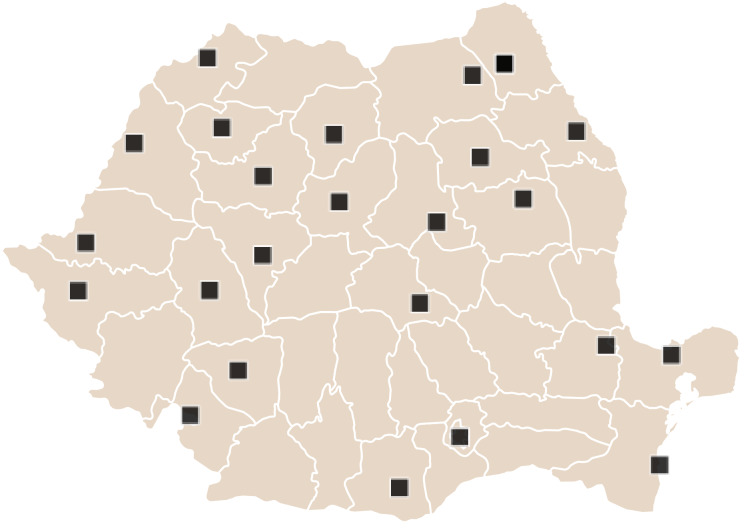
The location of the interviewed people on the Romania map.

We conducted an online survey with a convenience sample of Romanians from May to July 2022. A total of 417 Romanians from rural and urban areas from 24 counties (out of the total 41 counties and the municipality of Bucharest) were interviewed (Fig. 1). The average distance between the capital of Romania, Bucharest, and the 24 counties participating in the study is 283 km. The nearest county where people were interviewed is Teleorman, 80 km away, and the farthest county is Satu-Mare, 430 km away from the capital.

### Geographical coordinates

Romania is geographically located at the intersection of the 45th parallel North latitude and the 25th meridian East longitude, positioning it in the northern hemisphere. This country is situated in southeastern Central Europe, sharing its borders with the Black Sea to the east, Bulgaria to the south, Ukraine to the northeast, as well as Hungary, Serbia, and Moldova. It has a temperate continental climate and covers an area of 238,391 square kilometers. As of 2023, Romania’s population stood at 19,842,957, making it the seventh most populous member of the European Union (Maps of world.com, 2023).

### Environmental profile of Romania

Romania boasts a diverse environmental profile, encompassing rich biodiversity, the Carpathian Mountains, the Danube River, and the Black Sea coast, making it a country of significant ecological importance in Europe.

Biodiversity in Romania is protected by the EU Biodiversity Strategy for 2030, adopted in 2020, which aims to bring EU biodiversity on the road to recovery, establishing new objectives and governance mechanisms to ensure healthy and resilient ecosystems. This strategy contains several goals such as protecting over 30% of the EU’s land area and 30% of the maritime area, protecting at least one-third of the EU’s protected areas (forests) (Eurostat, 2023a).

The EU Forest Strategy for 2030, adopted in 2021, is part of a legislative package, “Prepare for 55”. The main objective of this strategy is to ensure healthier and more resilient forests to support biodiversity and climate goals, as forests are important carbon sinks, and their conservation is vital, helping climate neutrality (European Union Law, 2022).

Romania has a circular economy action plan at the national level. It also has sectoral strategies for plastics, textiles, construction, or waste management, but they are less comprehensive than in the European Union (Eurostat, 2023b). Measures must be adopted to increase the circular use of materials and waste management. The recycling rate of municipal waste continues to be low, with a percentage of 14%, compared to the European Union average of 48%.

### Country profile regarding health

According to the European Commission (2021), life expectancy in Romania increased by over 4 years between 2000 and 2019, but unfortunately, due to the impact of the COVID-19 pandemic, it temporarily decreased by 1.4 years in 2020.

Among the risky behaviors in terms of health among Romanians, the rate of alcohol consumption and unhealthy eating contribute to almost half of all deaths. Adult obesity is the lowest in the EU, and smoking prevalence among adults is slightly lower than the EU average. These risk factors are more common among men than among women, but overweight, obesity, and smoking among teenagers have risen steadily over the past two decades, being above the EU average. In the last decade, health spending in Romania increased by an average of 10.3%/year, but still remained at the second lowest level in the EU, both as a percentage of GDP and as a share *per capita* (European Commission, 2021).

The questionnaire had 21 questions/statements (see Supplemental File_Questionnaire). The questions were related to the awareness of MPs, the perceived health risk of MPs, the perceived environmental risk of MPs, the intensity of exposure to media narratives about the MPs impact on health and the environment, and the demographics (Table 1). Drawing upon pertinent literature (Deng et al., 2020; Soares et al., 2021; Qi et al., 2022) and theoretical frameworks that substantiate the integration of these demographic variables in environmental studies and media research enhance the justification for the inclusion of these demographic variables, showcasing their extensive examination and theoretical significance. The selected demographic variables (age, income, living area, gender, and education) are crucial to comprehensively assess the influence of media narratives on MPs, as they directly impact individuals’ awareness, attitudes, vulnerability, and behavior towards environmental issues and public health concerns. Kramm et al. (2022) found that the influence of media narratives on risk perception varied with gender and age. Health risk perception was higher for women with an increased knowledge of media narratives. Similarly, Garcia-Hidalgo et al. (2017) found that age and gender influenced MPs discharge. Different demographic groups might have varying awareness and knowledge about MPs due to their access to media sources and educational opportunities. Individuals with lower income and education levels may need more access to resources and information to protect themselves from exposure. Gender and educational levels predicted awareness of MPs pollution among Indian students (Dowarah, Duarah & Devipriya, 2022). Including these variables can help identify potential disparities and differential risk perceptions. Identifying which demographic groups might be responsive to particular media messages can inform communication efforts to raise awareness and promote sustainable behaviors.

**Table 1:** Investigated variables.

Investigated variables	Questionnaire question	Answer options
(1) Awareness of MPs	(1) Have you heard of MPs?	1 = Yes/0 = No
(2) Perceived health risk of MPs	(2) Are you concerned about the risks that MPs have on your health?	1 = Yes/0 = No
(3) Perceived environmental risk of MPs	(3) Are you concerned about the risks posed by MPs to the environment?	1 = Yes/0 = No
(4) Exposure intensity to media narratives	(4) Various narratives on MPs, highlighting the MPs impact on the environment and health, are presented in the media. Which of the following statements have you heard before? *Media narratives on health impact:* – “MPs cause cancer.” – “MPs cause respiratory diseases.” – “MPs cause intestinal diseases.” – “Ingestion of MPs can cause alteration of chromosomes, which leads to infertility.” *Media narratives on environmental impact:* – “MPs in the sea threaten fish stocks.” – “Animals die from the ingestion of MPs.” – “Leakage of harmful chemicals from MPs affects the soil.” – “MPs in soil limit the growth of plants.”	1 = I have never heard,…, 7 = I have heard daily/every few days*

**Note:**

* For the independent variables, we opted for a seven-point Likert scale, which offers several variants of options, increasing the probability of meeting the objective reality of people and allowing respondents to choose the exact option rather than the closest one (Dawes, 2008; Amicarelli et al., 2022).

The list of media narratives about the environmental impact (“Microplastics in the sea threaten fish stocks”, “Animals die from ingestion of microplastics”, “ Leakage of harmful chemicals from MPs affects the soil”, “Microplastics in soil limit the growth of plants”) and the first three media narratives about the health impact (“Microplastics cause cancer”, “Microplastics cause respiratory diseases”, and “Microplastics cause intestinal diseases”) were extracted from Kramm et al.’s (2022) work. To these, we added the media narrative “Ingestion of microparticles can cause alteration in chromosomes which lead to infertility” based on the MPs potential hazardous effects on humans reported by Sharma & Chatterjee (2017). Media narratives often transcend borders due to the interconnectedness of today’s media landscape. Narratives that acquire momentum inside a particular nation have the potential to disseminate across international borders rapidly. Therefore, if media outlets in other countries have covered MPs and their health and environmental implications, Romanian media would also be inclined to cover them. Additionally, the media tends to disseminate narratives widely covered in scientific research. Romanian media would likely report on these findings to inform their audience about global health and environmental issues.

The questionnaire was written in Romanian and implemented on an online platform (Isondaje). The questionnaire link was distributed *via* e-mail and on several social networks (*e.g*., Facebook, and Instagram). The demographic profile was described by gender, education, living area, age, and income (Table 2). The average age was 38.5 years, close to the average age in Romania of 42, 1 years, according to National Institute of Statistics of Romania (INSS) (2019). Women represent 67.9% of the sample; in Romania, the masculinity ratio on January 1, 2019, was 95 men to 100 women (National Institute of Statistics of Romania (INSS), 2019).

**Table 2:** Summary profile of the respondents.

Variable	Category
(1) Gender (% of the sample)	Female: 67.9%; Male: 32.1%; Not specified: 0%
(2) Education (completed level)	8 classes: 4.1%; 12 classes: 29.7%; university: 66.2%
(3) Living environment (% of the sample)	Urban area: 81.3%; Rural area: 18.7%
(4) Age (years; average)	38.5 years
(5) Monthly income/family	3,000 lei (600 Euros): 20.6%; 3,001–6,000 lei (601–1,200 Euros): 35.3%; 6,001–9,000 lei (1,201–1,800 Euros): 9,001–12,000 lei (1,801–2,400 Euros): 19.4%

A non-probabilistic snowball method was implemented to distribute the link of the questionnaire. Snowball sampling was a method that was widely applied in the pandemic context (Cohen & Arieli, 2011) when people were not very willing to be surveyed face-to-face, requiring minimal human resources. However, representation is not guaranteed, and it is vulnerable to sampling biases (*e.g*., risk of self-selection) (Amicarelli et al., 2022). The major drawback of snowball sampling is the lack of randomization, which can lead to sampling biases. To mitigate this, we implemented specific selection criteria: to have both men and women from urban and rural areas, to include people from all educational and income categories, of all age groups, and to cover at least half of Romania’s counties. These inclusion criteria balance the demographics and backgrounds of the participants, aiming to minimize systematic biases that might arise. Being aware that individuals who participate in snowball sampling may self-select into the study, potentially leading to biased results, researchers made efforts to communicate the importance of diverse perspectives and experiences in the study. They encouraged participants to refer others who might hold different viewpoints or backgrounds. Informed consent was asked of the participants, their answers were anonymous, and the scientific purpose of the online survey was explained at the beginning of the questionnaire text.

The dependent variables were coded as dichotomous variables. Therefore, we opted for binary logistic regression. Binary logistic regression was performed three times using SPSS to test:

(i) The relationship between media narratives on the impact of MPs (independent variables) and awareness of MPs (as dependent variable);

(ii) The relationship between media narratives on the impact of MPs (independent variables) and perceived health risks of MPs (dependent variable);

(iii) The relationship between media narratives on the impact of (independent variables) and the perceived environmental risks of MPs (dependent variable). Gender and age were included in the binary logistics as independent variables.

Binary logistic regression was run to identify what media narratives influenced MPs awareness and risk perception. Several outputs provided by SPSS, as part of the logistic regression, were considered in the analysis (Pallant, 2013). Thus, the Omnibus Tests is a “goodness of fit” test and shows how well the model performed. A Sig. value below 0.05 must be obtained. Hosmer and Lemeshow Test is another important indicator of the goodness of fit, and the obtained value must be higher than 0.05. The Cox & Snell R Square and the Nagelkerke R Square values indicate how much of the variation in the dependent variable (in our case, “Awareness of MPs”/“Perceived health risk of MPs”/“The perceived environmental risks of MPs”) is explained by the model. The values can range between 0 and 1. The *p* values less than 0.05 associated with each of the independent variables signal which of them contributes significantly to the model’s predictive ability. The sign of B values (Tables 3–5), indicate the direction of the relationship between the independent and dependent variables (which independent variables increase the likelihood of a “yes” answer and which decrease it). The present study coded the dichotomous variables with 0 = no and 1 = yes. Therefore, positive B values mean that an increase in the independent variable score (higher media exposure) is associated with an increase in the probability of reporting awareness of MPs and perceived risk of MPs. The Exp(B) column (in the same tables) is the odds ratio (OR) for each independent variable. The odds ratio shows the increase in odds of being in one outcome category when the value of the predictor increases by one unit (or the decrease if the ratio is less than one) (Tabachnick & Fidell, 2007). The analyses were performed with Excel and SPSS.

**Table 3:** Results of the binary logistic regression analysis for the impact of selected variables on the awareness of MPs (only variables with prediction power are included in the table).

Dependent variable: Awareness of MPs						
Independent variables	B	S.E.	Wald	df	Sig.	Exp(B)
(1) Microplastics in the sea threatened fish stocks	0.297	0.091	10.649	1	0.001	1.346
(2) Age	0.040	0.008	25.750	1	0.000	0.814

**Note:**

B, the regression coefficient; S.E., standard error; Wald, wald statistic; df, the degree of freedom; *p*, significance; Exp(B), the odds ratio.

**Table 4:** Results of the binary logistic regression analysis for the impact of selected variables on the perceived health risk of MPs (only variables with prediction power are included in the table).

Dependent variable: Perceived health risk of MPs						
Independent variables	B	S.E.	Wald	df	Sig.	Exp(B)
(1) Leakage of harmful chemicals from MPs affects the soil	0.426	0.174	5.991	1	0.014	1.531

**Note:**

B, the regression coefficient; S.E., standard error; Wald, wald statistic; df, the degree of freedom; *p*, significance; Exp(B), the odds ratio.

**Table 5:** Results of the binary logistic regression analysis for the impact of selected variables on the perceived environmental risk of MPs (only variables with prediction power are included in the table).

Dependent variable: The perceived environmental risks of MPs						
Independent variable	B	S.E.	Wald	df	Sig.	Exp(B)
(1) Leakage of harmful chemicals from MPs affects the soil	0.544	0.192	8.030	1	0.005	1.723
(2) Microplastics cause respiratory diseases	0.450	0.194	5.378	1	0.020	1.568

**Note:**

B, the regression coefficient; S.E., standard error; Wald, wald statistic; df, the degree of freedom; *p*, significance; Exp(B), the odds ratio.

## Results

### Media narratives of MPs influence the awareness of MPs

Omnibus Tests of Model Coefficients showed an overall indication of how well the proposed model performed. In our case, the Sig. value was 0.000 (*p* < 0.0005). The chi-square value was 85.66 with 10 degrees of freedom (Table A1). The chi-square value for the Hosmer-Lemeshow Test was 12.766 with a significance level of 0.120.

The value was higher than 0.05, therefore, indicating support for the model (Table A2). The Cox & Snell R Square and the Nagelkerke R Square values indicated the amount of variation in the dependent variable (“Awareness of MPs”) explained by the model (Table A3). For this first binary regression, the two values were 0.186 and 0.255, suggesting that between 19% and 25% of the variability in the “Awareness of MPs” was explained by this set of variables. The results of the first binary logistic regression showed that two variables contribute significantly to the prediction power of the model. The factors that influence the awareness of MPs are the age (*p* = 0.000) and the media narrative “Microplastics in the sea threaten fish stocks” (*p* = 0.001) (Table 3), and their influence is as follows. When the frequency of exposure to the media narrative “Microplastics in the sea threatens fish stocks” increases, the probability of reporting awareness of MPs increases. An increase in age represents a higher probability of reporting awareness of MPs.

### Media narratives of MPs influence the perceived health risks of MPs

The outputs generated by SPSP indicated support of the model. Thus, the Sig. value of the Omnibus Tests of Model Coefficients was 0.000. The chi-square value was 53.165 with 10 degrees of freedom (Table A4). The chi-square value for the Hosmer-Lemeshow Test was 8.062 with a significance level of 0.427 (Table A5). The Cox & Snell R Square and the Nagelkerke R Square values indicated that between 12% and 27% of the variability in “The perceived health risks of MPs” was explained by the media narrative “Leakage of harmful chemicals from MPs affects the soil” (Table A6).

The factor that influenced the perceived health risk of MPs was the media narrative “Leakage of harmful chemicals from MPs affects the soil” (*p* = 0.014) (Table 4). When the frequency of exposure to this media narrative increases, the probability of reporting the perceived health risk of MPs increases.

### Media narratives of MPs influence the perceived environmental risks of MPs

The Omnibus Tests of Model Coefficients showed that Sig. the value was 0.000 (this *p* < 0.0005). The chi-square value was 57.709 with 10 degrees of freedom (Table A7). The chi-square value for the Hosmer-Lemeshow Test was 11.808 with a significance level of 0.160 (Table A8), indicating support for the model. The Cox & Snell R Square and the Nagelkerke R Square values showed that between 13% and 30% of the variability in the dependent variable (“The perceived environmental risks of MPs”) was explained by two variables (Table A9). Thus, the media narratives “Leakage of harmful chemicals from MPs affects the soil” (*p* = 0.005) and “Microplastics cause respiratory diseases” (*p* = 0.020) influenced the “Perceived environmental risks of MPs” (Table 5). This means that when the frequency of exposure to the media narratives “Leakage of harmful chemicals from MPs affects the soil” and “Microplastics cause respiratory diseases” increases, the probability of reporting perceived environmental risks of MPs increases. Gender and age did not influence the awareness of MPs, the perceived health risks of MPs, or the perceived environmental risks of MPs.

An overview of all variables included in regression models and their level of significance, for all the hypothesis, is presented in Table A10. An optional question asked people about other types of information they received from the media about MPs. Several answers were offered, and some of them will be discussed in the next section.

### Respondents’ responses on the risk of MPs to the environment and human health

Some of the answers received from respondents regarding the open question “Do you have other media information about MPs?” are discussed further. Several responses linked the MPs to vulnerable groups, mainly children. One respondent said that “MPs affect to a greater extent the health of vulnerable groups (children, pregnant women, *etc*.)”. Due to their physical characteristics, MPs are passed through the entire food chain, so they can primarily affect vulnerable groups such as pregnant women, children, *etc*. Some scientific papers report on this and show how early exposure to MPs can occur through the placenta, bottles, breastfeeding by the mother, infant formula, and plastic toys (Song et al., 2021; Ragusa et al., 2022). Some studies revealed that millions of MPs particles are released when infant formulas are prepared in polypropylene (PP) bottles (Li et al., 2020). Zhang et al. (2021) investigated the concentrations of polyethylene terephthalate (PET) and polycarbonate (PC) for six infants and found higher concentrations of PET in the feces of the infants compared to those of adults.

Another person believed that “Each of us has enough plastic in our body to make a card”. Other people wrote about the sources of MPs or their impact, like “There are MPs in cigarettes”, “There are MPs in lawns”, and “Because of this, hundreds of marine species die every year”. Another person stated he did not do much about MPs since there were hotter topics to be debated (“I do not do much because it is not a mainstream topic such as climate change, which is often debated in the media”). Indeed, they are hot topics debated much more often than MPs. While topics like climate change receive more attention, it’s essential not to overlook the need for vulnerability assessments and the role of risk perception in addressing MPs pollution. Therefore, authorities have to assess the risk of MPs by taking into account factors such as the level of pollution, how extensively the pollution has spread, the pathways and movement of pollutants, and conducting evaluations for ecological and health risks to the environment (Ozunu et al., 2009).

One interviewed person said he heard from media sources about MPs on the lawn. According to Habib et al. (2022), artificial turf can be a significant MPs source. The amount of MPs released is highly dependent on the type of turf, its age, and how it is used.

Another person wrote that he heard that there would be specific legislation for the MPs.

Globally, the continuous increase in plastic waste is countered by using various strategies such as introducing bans and limits on plastic items as well as plastic materials, continuously promoting recycling strategies, increasing the percentage of plastic recovery and encouraging activity volunteers, clean-up actions, and last but not least, raising public awareness, which was also tracked through our survey. Plastic materials contain dangerous additives and can adsorb environmental pollutants (heavy metals and, respectively, persistent organic contaminants). For this reason, plastic materials’ toxicity to the environment and human health is not only related to the polymer chains.

Recent concerns about the environment and human health worldwide have led to decisions in several European Union member states that have introduced or proposed a national ban on the intentional use of MPs in food products intended for broad consumption. According to the Parlamentul European (2018), every year, approximately 42,000 tons of MPs end up in the environment after using products containing them.

In 2002, in Ireland, according to Convery, McDonnell & Ferreira (2007), a plastic bag tax was introduced that reduced the consumption of these items by more than 90%, leading to a decrease in both waste and negative effects on the environment. Dikgang, Leiman & Visser (2012) highlighted that the South African government implemented legislation on the use of single-use polyethylene shopping bags. The US federal government introduced the Germ-Free Water Act of 2015. This law aims to ban the manufacture and sale of microbeads in cosmetic products starting in July 2018. Canada passed legislation to ban the production of microbeads in June 2017. Australia established a group to seek voluntary agreement from the industry to phase out microbeads from personal care, cosmetic, and cleaning products (Environment Protection Authority, 2016).

**Figure 2:**
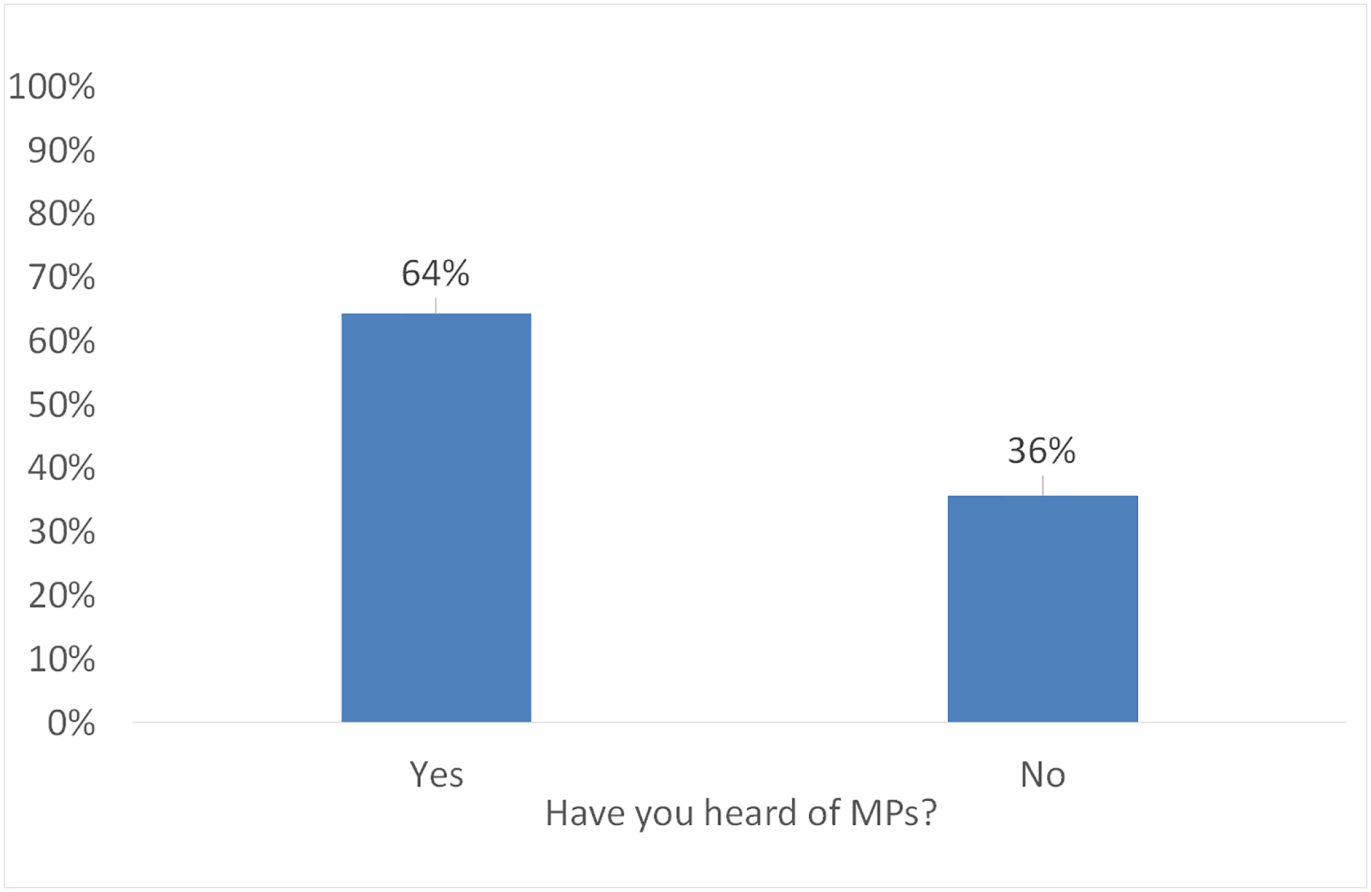
Awareness of MPs.

When we compare certain findings from our study, like the response to the question “Are you aware of microplastics (MPs)?”, we see that in Romania, over 60% of respondents were aware of MPs, whereas in Germany, the majority (80%) had knowledge of them (Kramm et al., 2022), (Fig. 2: Awareness of MPs). This increased awareness of the risks associated with MPs in both countries can be attributed to recurring media coverage. Additionally, we found similar results in both Romania and Germany, with respondents in both countries expressing more concern about health risks than environmental risks.

## Discussion

### The influence of media narratives on the awareness of MPs

Regarding the first hypothesis, we found that the media narrative “Microplastics in the sea threaten fish stocks” influenced the MPs risk awareness. Additionally, the demographic variable “age” influenced the awareness. Therefore, the older the age, the greater the awareness related to the risk of MPs. As the frequency of media narratives increases, the likelihood of reporting awareness of this type of MPs risk increases.

An explanation of these results could stand in the popularized topic of the impact of MPs on marine ecosystems (Digi24, 2020; Engle, 2022; Matei, 2021; Michel, 2022). This marine pollution is entirely caused by human behavior and decisions (Pahl, Wyles & Thompson, 2017). Keller & Wyles (2021) consider that knowledge of how the public perceives the pollution of MPs and its sources of information are key steps in identifying solutions to address the MPs impact on the aquatic environment. They point to the media as one of the vehicles that can shape public opinion and promote pro-environmental behaviors. Being highly mediatized, the public perception of marine pollution has changed in the last decade. For example, in 2009, only 3.1% of the participants in a restricted sample from the United Kingdom mentioned marine litter as a pressing problem (Fletcher et al., 2009). A few years later, in 2013, it was found that 50% of the participants in a survey were aware of the term “microplastics” (GESAMP, 2015).

According to the Plastics Europe Association of Plastics Manufacturers (2015), plastic represents approximately 80% to 85% of marine litter. Furthermore, plastic has indirectly become the segment with the highest share, especially in municipal waste generation, due to the increase in production by 176 times (from 1.7 million tons in the 1950s to around 300 million tons in 2014). Therefore, the major problem is not only marine litter due to plastic, as MPs pollution starts in the soil and ends up in the seas and oceans.

### Perceived health risk of MPs

The perceived health risk of MPs, with the highest weighting, was related to the dependent variable “Leakage of harmful chemicals from MPs affects the soil”. Although science and media have reported and popularized plastic pollution of aquatic resources more frequently, a series of studies also confirmed the presence of MPs in the soil. Therefore, Tudor et al. (2019) considers soil pollution with MPs to be increasing aggressively and estimates it to be up to 23 times higher than water pollution. The accumulation of MPs in the soil by leaks affects the soil biota, the plastic material is assimilated to the organic matter and can persist for several hundreds of years (Tudor et al., 2019). The results of the present study showed that Romanians perceive the risk to human health related to MPs in the soil.

Why do Romanians consider MPs particles in the soil a risk to human health? We can bring some arguments and answers from our questionnaire. For example, one of the respondents emphasized that “it takes hundreds of years for the soil to regenerate following MPs pollution”. Another one claimed that “the problem in this situation would also be related to the existing means, at least in the city where he lives/related to recycling (there were several reports that indicated the fact that recycled materials still end up in the landfill, and they are not recycled as they should be), thus further contaminating the soil”. Urgent measures must be taken so that these plastic materials are properly recycled so that they do not end up in the soil or the atmosphere, through decomposition causing severe diseases in everything that means life. One of the statements read “I watch almost weekly short reports about environmental pollution and the effects which it has on the body”.

Unfortunately, not much information is presented in the mass media so that most of the population is aware of the risks for human health. These MPs particles, through the food chain, once they reach the soil produce, in addition to the environmental risk, a risk to human health. According to Vethaak & Legler (2021), this food chain pollution is a “Trojan horse” type of pollution connecting both to the environment and ultimately to human health. Igalavithana et al. (2022) considers MPs vectors for other soil contaminants, such as potentially toxic elements (PTE) and organic pollutants. Rahman et al. (2021) argue that further research is needed to quantify the effects of MPs on human health and their pathogenesis based on their initial search that resulted in 17,043 published articles and literature documents. After a comprehensive review, he identified 129 publications based on the risk of MPs particles on human health.

Therefore, countermeasures are explored and implemented worldwide, including policies and governance-based approaches aimed at the circular economy to reduce environmental contamination by MPs. Sustainable thinking generates valuable information for marketing entities that will influence consumers when making purchasing decisions. In a study by Choi et al. (2022) investigated the recognition and attitude toward MPs and zero waste, it was shown that the recognition of the adverse effect of MPs on health was a significant factor in promoting zero waste awareness and behavior.

Consequently, from a practical perspective, the findings of this study should attract the attention of producers, governments, and the media and come up with solutions to reduce this type of risk through different campaigns that will raise red flags. Farmers who pollute the soil with MPs due to plastic film need legislation and financial aid to replace the protective plastic film with an environmentally friendly one. A compostable plastic film made of polylactic acid (PLA) material that is usually obtained from natural products such as sugar beet, corn, or other plants could reduce the perceived health risk of MP over time. This type of compostable plastic used correctly and respecting the entire recycling cycle, can reduce the health and environmental impact of MP. People usually look for food quality indicators related to health and environmental protection (Petrescu et al., 2022; Petrescu, Petrescu-Mag & Burny, 2015), which implies unpolluted soil to produce healthy food.

Janoušková et al. (2020) presents original findings that contribute to international research on the influence of education and mass media on people’s understanding of environmental sustainability and their perception of health risks associated with MPs particles. Pro-environmental campaigns conducted by NGOs and public entities can serve as a solution to enhance awareness. Finally, the solutions should not only help reduce soil pollution. According to Adji et al. (2022), in addition to soil and fauna, the concentrations of MPs increase because of the trophic chain, finally indicating possible biomagnification, that is a risk of contamination with MPs, ultimately related to both the environment and health.

### The perceived environmental risks of MPs

The perception of risk significantly impacts how society reacts to a specific risk, such as MPs.

According to Ustohalova (2011), environmental risk refers to the probability of affecting the natural environment, such as ecosystems, biodiversity, air, water, soil, and other ecological components, resulting from human activities or natural events. Over time, there have been cases where a mismatch between the actual risk and the perception of it has led to bad decisions (Van der Weijden et al., 2007). Therefore, the chances of correctly reacting are higher when the perceptions of risks are formed based on correct information. Media narratives about pro-environmental behavior regarding MPs exposure could overcome unfavorable behaviors alongside published data about plastic waste management, new strategies, and interventions that tackle plastic pollution (Maran & Begotti, 2021).

In the present study, the variable “Leakage of harmful chemicals from MPs affects the soil” had the highest impact on perceived environmental risks of MPs among the respondents, followed by “MPs causing respiratory diseases”. According to Huang et al. (2022) and Wojnowska-Baryła, Bernat & Zaborowska (2022), landfills are the main reservoirs of MPs. In the EU, in 2020, 31% of the total EU waste was landfilled (Eurostat, 2022). Romania is third after Malta and Greece, with more than 70% of waste being landfilled (European Environmental Agency, 2022).

To justify that narrative “MPs causing respiratory disease” can also be considered as a risk for the environment, we bring to the fore the argument for a holistic perspective on health risks. Thus, we consider that addressing human health independently of environmental concerns, or *vice versa*, can lead to incomplete solutions. MPs-induced respiratory diseases serve as a compelling example of how environmental degradation directly impacts human health. Thus, it is imperative to adopt a comprehensive approach to address these challenges effectively. MPs in the environment not only directly threaten human health but also indirectly jeopardize ecosystems and overall environmental integrity. Consequently, addressing the narrative of “Microplastics causing respiratory disease” is relevant not only for public health but also for environmental protection. Furthermore, the presence of MPs in the environment, particularly if they are associated with respiratory diseases or other health risks, can indirectly affect human activities such as fishing, tourism, and agriculture. When these activities rely on a healthy environment, a decline in environmental quality due to MPs contamination can lead to economic and societal consequences. Considering the potential synergistic effects, if someone is already exposed to air pollution due to MPs and then encounters additional pollutants, the combined impact on respiratory health can be significantly worse than addressing each pollutant separately. This underscores the importance of considering the interconnectedness of environmental and health risks to formulate comprehensive strategies for safeguarding both our well-being and the environment.

### Limitations

Many media narratives highlighted the impact of MPs on the respiratory tract (Yeung, 2021; Toma, 2022), and the atmosphere as a medium of MP dispersion and transportation (Jarosz et al., 2022). In Paris, MPs were detected in atmospheric precipitation, according to Prata (2018). Due to their small size, these particles can be inhaled and cause damage to the respiratory system, depending on their properties (Mintenig et al., 2019; An et al., 2022). Workers exposed to plastic fibers and particles were diagnosed with respiratory dyspnea and interstitial inflammatory problems (Lu et al., 2022). This mediatized topic could explain why the variable “MPs causing respiratory diseases” influenced the perceived environmental risk.

Several limitations of the study are presented in the following. A representative sample at the country level would have helped extrapolate the results to the entire population. The sample was not gender-balanced, since women represented 67.9% of the sample, and they are known to experience higher levels of media (especially social media) exposure and addiction (Su et al., 2020). Moreover, the risk of Internet surveys is that the respondents cannot be contacted, and thus receive further explanations. They can complete the questionnaire by quickly clicking on each question without reading carefully. Additionally, future research could investigate a larger number of media narratives dedicated to MPs.

## Conclusions

The existing literature lacks comprehensive information regarding the health and environmental impacts of MPs. Nevertheless, the available scientific data raise concerns about this global issue. Recognizing the influential role of media in shaping public attitudes and behaviors, our study investigates whether media narratives influence awareness and perceptions of health and environmental risks associated with MPs.

Our findings indicate that age and specific media narratives significantly influence the awareness of MPs. Specifically, an increase in the frequency of media reports about “MPs in seawater threatening fish stocks” correlates with higher awareness levels. Interestingly, our research shows that individuals are more concerned about the health risks posed by MPs than the environmental risks. These results offer valuable insights for NGOs, public authorities, and decision-makers involved in environmental protection and health communication campaigns. Early childhood education initiatives can also play a role in increasing awareness of MPs. By intensifying and promoting information, education, and communication services related to environmental protection, we have the potential to change people’s perceptions, attitudes, and behaviors. This can ultimately lead to reduced plastic consumption, proper disposal, and decreased pollution risks associated with MPs, benefiting both human health and the environment.

Integrated waste management practices and government policies on plastic use represent sustainable approaches to mitigating MPs pollution. Mass media plays a pivotal role in shaping people’s behaviors, values, and attitudes. Therefore, it is crucial for political decision-makers and communicators (including researchers and mass media) to focus on practical and effective solutions that empower individuals to take daily actions and make informed decisions that contribute to reducing MPs pollution. According to Worm et al. (2017), governments should consider the reclassification of plastics based on new evidence regarding their impact on human health and the environment. This could lead to the establishment of a new international convention aimed at addressing plastic, MPs, and nanoplastic pollution.

The field of MPs research is gaining momentum and public interest. However, it remains an area that lacks extensive coverage in reputable scientific journals, underscoring the need for pioneering studies. In Romania, there are notable gaps in MPs research, spanning from sampling to laboratory investigations and beyond. Decision-makers should prioritize and invest in this area, given that MPs pose risks to all four environmental factors: soil, air, water, and biodiversity. We hope that the outcomes of our analysis will contribute to the advancement of research in the field of MPs. We aspire to serve as a catalyst for renewed international research efforts in this critical area.

## Supplemental Information

10.7717/peerj.16338/supp-1 Supplemental Information 1 Raw data.

10.7717/peerj.16338/supp-2 Supplemental Information 2 Results of statistical analyses.

10.7717/peerj.16338/supp-3 Supplemental Information 3 Romanian version of the questionnaire.

10.7717/peerj.16338/supp-4 Supplemental Information 4 English version of the questionnaire.

## Additional Information and Declarations

### Data Availability

The following information was supplied regarding data availability: The respondents’ answers to the questionnaire are available in the Supplemental Files.
