# Supplementary material for: The influence of media narratives on microplastics risk perception
Source: PeerJ. 2023 Nov 2;11:e16338. doi: 10.7717/peerj.16338 (PMC10625762; doi:10.7717/peerj.16338)
Supplement: Supplemental Information 2 [file peerj-11-16338-s002.docx]

**Appendix. Results of statistical analyses**

| **Table 1A:**  **Omnibus Tests** | | | | |
| --- | --- | --- | --- | --- |
|  | | **Chi-square** | **df** | **Sig.** |
| Step 1 | Step | 85.665 | 10 | .000 |
|  | Block | 85.665 | 10 | .000 |
|  | Model | 85.665 | 10 | .000 |

| **Table 2A:**  **Hosmer and Lemeshow Test** | | | |
| --- | --- | --- | --- |
| **Step** | **Chi-square** | **df** | **Sig.** |
| 1 | 12.766 | 8 | .120 |

| **Table 3A:**  **Cox & Snell R Square and Nagelkerke R Square values** | | | |
| --- | --- | --- | --- |
| **Step** | **-2 Log likelihood** | **Cox & Snell R Square** | **Nagelkerke R Square** |
|  |  |  |  |
| 1 | 457.984^a^ | .186 | .255 |
| Note: Estimation terminated at iteration number 4 because parameter estimates changed by less than .001. | | | |

| **Table 4A:**  **Omnibus Tests** | | | | |
| --- | --- | --- | --- | --- |
|  | | **Chi-square** | **df** | **Sig.** |
| Step 1 | Step | 53.165 | 10 | .000 |
|  | Block | 53.165 | 10 | .000 |
|  | Model | 53.165 | 10 | .000 |

| **Table 5A:**  **Hosmer and Lemeshow Test** | | | |
| --- | --- | --- | --- |
| **Step** | **Chi-square** | **df** | **Sig.** |
| 1 | 8.062 | 8 | .427 |

| **Table 6A:**  **Cox & Snell R Square and Nagelkerke R Square values** | | | |
| --- | --- | --- | --- |
| **Step** | **-2 Log likelihood** | **Cox & Snell R Square** | **Nagelkerke R Square** |
| 1 | 192.002^a^ | .120 | .269 |
| Note: Estimation terminated at iteration number 6 because parameter estimates changed by less than .001. | | | |

| **Table 7A:**  **Omnibus Tests of Model Coefficients** | | | | |
| --- | --- | --- | --- | --- |
|  | | **Chi-square** | **df** | **Sig.** |
| Step 1 | Step | 57.709 | 10 | .000 |
|  | Block | 57.709 | 10 | .000 |
|  | Model | 57.709 | 10 | .000 |

| **Table 8A:**  **Hosmer and Lemeshow Test** | | | |
| --- | --- | --- | --- |
| **Step** | **Chi-square** | **df** | **Sig.** |
| 1 | 11.808 | 8 | .160 |

| **Table 9A:**  **Cox & Snell R Square and Nagelkerke R Square values** | | | |
| --- | --- | --- | --- |
| **Step** | **-2 Log likelihood** | **Cox & Snell R Square** | **Nagelkerke R Square** |
| 1 | 173.022^a^ | .129 | .304 |
| Note: Estimation terminated at iteration number 6 because parameter estimates changed by less than .001. | | | |

| **Table 10A:**  **Overview of all variables included in regression models and their level of significance** | | | |
| --- | --- | --- | --- |
| **Independent variable** | **Significance*** | | |
|  | **H1**** | **H2**** | **H3**** |
| “MPs cause cancer.” | .885 | .154 | .300 |
| “MPs cause respiratory diseases.” | .472 | .149 | .020* |
| “MPs cause intestinal diseases.” | .505 | .214 | .802 |
| “Ingestion of MPs can cause alteration of chromosomes, which leads to infertility.” | .055 | .110 | .959 |
| “MPs in the sea threaten fish stocks.” | .001* | .190 | .772 |
| “Animals die from the ingestion of MPs.” | .661 | .489 | .817 |
| “Leakage of harmful chemicals from MPs affects the soil.” | .687 | .014* | .005* |
| “MPs in soil limit the growth of plants.” | .212 | .171 | .091 |
| Gender | .814 | .104 | .074 |
| Age | .000* | .414 | .299 |
| * The variables with a significant prediction power (p < .05) are marked with “*”.  ** H1. Media narratives of MPs influence the awareness of MPs; H2. Media narratives of MPs influence the perceived health risks of MPs; H3. Media narratives of MPs influence the perceived environmental risks of MPs. | | | |
