# Supplementary material for: The influence of media narratives on microplastics risk perception
Source: PeerJ. 2023 Nov 2;11:e16338. doi: 10.7717/peerj.16338 (PMC10625762; doi:10.7717/peerj.16338)
Supplement: Supplemental Information 3 [file peerj-11-16338-s003.docx]

| 1. Ați auzit de microplastic (MPs)? | Da/Nu |
| --- | --- |
| 1. MPs sunt împărțite în două tipuri: primare și secundare. *MPs primare* includ microbilele găsite în produsele de îngrijire personală, peleții de plastic utilizați în producția industrială și fibrele de plastic utilizate în textilele sintetice (de exemplu, nailon). Particulele de MPs primare intră în mediu printr-o serie de canale, cum ar fi utilizarea diverselor produse (de exemplu, produsele de îngrijire personală care intră în sistemele de apă uzată din gospodării), pierderea neintenționată de material plastic în timpul producției, transportului sau abraziunea în timpul spălării (de exemplu, spălarea îmbrăcămintei realizată din materiale sintetice). *MPs secundare* se formează din descompunerea materialelor plastice mai mari; acest lucru se întâmplă de obicei atunci când materialele plastice mai mari suferă intemperii, prin expunerea lor la acțiunea valurilor, vântului și radiațiile ultraviolete. | Nu se cere răspuns. |
| 1. Vă îngrijorează riscurile expunerii la MPs asupra sănătății dumneavoastră? | Da/Nu |
| 1. Vă îngrijorează consecințele pe care poluarea cu MPs le are asupra mediului natural? | Da/Nu |
| 1. Care dintre următoarele informații vă sunt cunoscute din mass media?    1. MPs provoacă boli canceroase.    2. MPs provoacă boli respiratorii.    3. MPs provoacă boli intestinale.    4. Ingestia de MPs poate provoca alterarea cromozomilor care duce la infertilitate.    5. MPs din mare amenință stocurile de pește.    6. Animalele mor din cauza ingerării MPs.    7. Substanțele chimice nocive ce se scurg din MPs afectează solul.    8. MPs din sol limitează creșterea plantelor. | 1=Nu am auzit niciodată ….. 7= Am auzit aproape zilnic |
| 1. Alte informații obținute din mass-media despre MPs | Întrebare deschisă cu răspuns opțional |
| 1. Genul | M/F/N |
| 1. Vârsta | Întrebare deschisă |
| 1. Locul de reședință | Urban  Rural |
| 1. Județul | Întrebare deschisă |
| 1. Educația (cel mai inalt nivel absolvit sau în curs de desfăsurare) | 8 clase  12 clase  Studii univesitare |
| 1. Venitu mediu lunar net pe întreaga familie | maximum 3000 lei / lună  3001- 6000 lei / lună  6001- 9000 lei / lună  9001- 12000 lei / lună |
| 1. Vă rugăm să folosiți spațiul de mai jos dacă doriți să ne spuneți/comentați ceva. |  |
