# Supplementary material for: The influence of media narratives on microplastics risk perception
Source: PeerJ. 2023 Nov 2;11:e16338. doi: 10.7717/peerj.16338 (PMC10625762; doi:10.7717/peerj.16338)
Supplement: Supplemental Information 4 [file peerj-11-16338-s004.docx]

| 1. Have you heard of microplastics (MPs)? | Yes/No |
| --- | --- |
| 1. MPs are divided into two types: primary and secondary. *Examples of primary MPs* include microbeads found in personal care products, plastic pellets (or nurdles) used in industrial manufacturing, and plastic fibres used in synthetic textiles (e.g., nylon). Primary MPs enter the environment directly through any of various channels – for example, product use (e.g., personal care products being washed into wastewater systems from households), unintentional loss from spills during manufacturing or transport, or abrasion during washing (e.g., laundering of clothing made with synthetic textiles). *Secondary MPs* form from the breakdown of larger plastics; this typically happens when larger plastics undergo weathering, through exposure to, for example, wave action, wind abrasion, and ultraviolet radiation from sunlight. | No answer requested. |
| 1. Are you concerned about the risks that MPs have on your health? | Yes/No |
| 1. Are you concerned about the risks posed by MPs to the environment? | Yes/No |
| 1. Which of the following information do you know from the media?    1. MPs cause cancer.    2. MPs cause respiratory diseases.    3. MPs cause intestinal diseases.    4. Ingestion of MPs can cause alteration of chromosomes, which leads to infertility.    5. MPs in the sea threaten fish stocks.    6. Animals die from the ingestion of MPs.    7. Leakage of harmful chemicals from MPs affects the soil.    8. MPs in soil limit the growth of plants. | 1=I’ve never heard ………….. 7= I’ve heard daily/ every few daily |
| 1. Other information obtained from the mass media about MPs: | Open question with optional answer |
| 1. Gender | M/F/N |
| 1. Age | Open question |
| 1. Place of residence | Urban  Rural |
| 1. County | Open-ended question |
| 1. Education (highest level completed or in progress) | 8 classes  12 classes  university studies |
| 1. Average monthly net income for the entire family | maximum 3000 lei / month  3001- 6000 lei / month  6001- 9000 lei / month  9001- 12000 lei / month |
| 1. Please use the space below if you want to tell us/comment something |  |
